# 4-Nitro­phenyl methacrylate

**DOI:** 10.1107/S1600536808025130

**Published:** 2008-08-13

**Authors:** Yun-Hua Xu, Fanqi Qu

**Affiliations:** a School of Science, Beijing Jiaotong University, Beijing 100044, People’s Republic of China; b College of Chemistry and Molecular Sciences, Wuhan University, Wuhan, Hubei 430072, People’s Republic of China

## Abstract

The title compound, C_10_H_9_NO_4_, was obtained serendipitously during the preparation of benzyl cyclo­hexyl­carbamate. The mol­ecule consists of two approximately planar parts, the nitro­phenyl ring and the rest of the non-H atoms, with a dihedral angle of 55.05 (6)° between the two segments. The crystal structure is stabilized by weak C—H⋯O inter­actions and π stacking [3.753 (1) Å] along the *b* axis.

## Related literature

For related literature, see: Banks *et al.* (1977 ▶); Hwang *et al.* (2007 ▶); Li *et al.* (2007 ▶); Otsu *et al.* (1968 ▶); Tang *et al.* (2007 ▶).
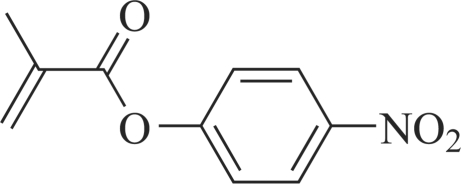


## Experimental

### 

#### Crystal data

C_10_H_9_NO_4_
*M* *_r_* = 207.18
Monoclinic, 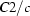

*a* = 24.491 (6) Å
*b* = 3.753 (1) Å
*c* = 23.428 (6) Å
β = 116.98 (1)°
*V* = 1919.0 (9) Å^3^
*Z* = 8
Mo *K*α radiation
μ = 0.11 mm^−1^
*T* = 90.0 (2) K
0.30 × 0.10 × 0.04 mm

#### Data collection

Nonius KappaCCD diffractometer
Absorption correction: multi-scan (*SCALEPACK*; Otwinowski & Minor, 1997 ▶) *T*_min_ = 0.967, *T*_max_ = 0.996
3936 measured reflections
2193 independent reflections
1380 reflections with *I* > 2σ(*I*)
*R*_int_ = 0.049

#### Refinement

*R*[*F* ^2^ > 2σ(*F* ^2^)] = 0.053
*wR*(*F* ^2^) = 0.145
*S* = 1.04
2193 reflections
137 parameters
H-atom parameters constrained
Δρ_max_ = 0.32 e Å^−3^
Δρ_min_ = −0.28 e Å^−3^

### 

Data collection: *COLLECT* (Nonius, 2002 ▶); cell refinement: *DENZO-SMN* (Otwinowski & Minor, 1997 ▶); data reduction: *DENZO-SMN*; program(s) used to solve structure: *SHELXS97* (Sheldrick, 2008 ▶); program(s) used to refine structure: *SHELXL97* (Sheldrick, 2008 ▶); molecular graphics: *XP* in *SHELXTL* (Sheldrick, 2008 ▶); software used to prepare material for publication: *SHELXL97* and local procedures.

## Supplementary Material

Crystal structure: contains datablocks global, I. DOI: 10.1107/S1600536808025130/fl2212sup1.cif

Structure factors: contains datablocks I. DOI: 10.1107/S1600536808025130/fl2212Isup2.hkl

Additional supplementary materials:  crystallographic information; 3D view; checkCIF report

## Figures and Tables

**Table 1:** Hydrogen-bond geometry (Å, °)

*D*—H⋯*A*	*D*—H	H⋯*A*	*D*⋯*A*	*D*—H⋯*A*
C7—H7⋯O1^i^	0.95	2.41	3.130 (2)	133
C1—H1*B*⋯O4^ii^	0.95	2.64	3.546 (3)	159
C2—H2*A*⋯O3^ii^	0.98	2.68	3.611 (2)	159
C9—H9⋯O4^iii^	0.95	2.46	3.282 (2)	145

Symmetry codes: (i) 
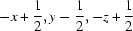
; (ii) 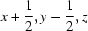; (iii) 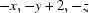.

**Fig. 1.**
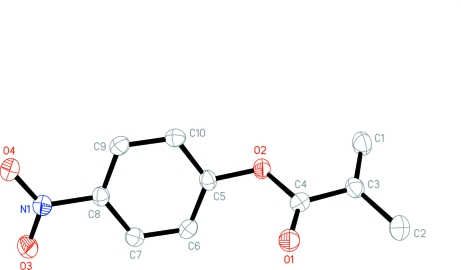
The molecular structure of (I), with displacement ellipsoids drawn at the 50% probability level (arbitrary spheres for the H atoms).

**Fig. 2.**
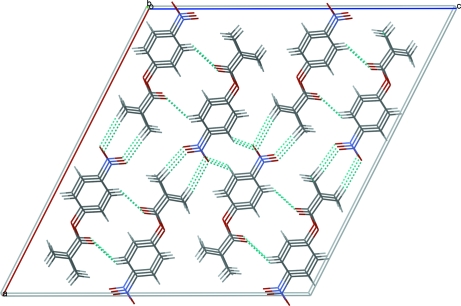
A packing diagram of (I) shown looking down the b axis.

## supplementary crystallographic information

### Comment

The title compound (I) is an important building block in the preparation of functional block polymers (Tang, *et al.* 2007; Hwang, *et al.* 2007; Li, *et al.* 2007). Although it has been widely used as a monomer in polymerization reactions for a long time (Otsu, *et al.* 1968), the crystal structure, as far as we know, has never been reported before.

Traditonally, (I) has been synthesized by refluxing methacryloyl chloride and *para*-nitrophenol (Banks, *et al.* 1977). Here it was obtained unexpectedly during an attempt to make benzyl cyclohexylcarbamate as described in the experimental section.

The asymmetric unit of (I) (Fig. 1) contains one molecule and bond lengths and angles are within normal ranges. The molecule consists of two approximately planar parts: the nitrophenyl ring and the rest of the non-hydrogen atoms (dihedral angle between the two segments is 55.05 (6)°). The nitro group is nearly coplanar with the phenyl ring as indicated by the torsion angle O3-N1-C8-C7 of  -7.48 °. The remaining non-hydrogen atoms are almost coplanar as suggested by the torsion angle C2-C3-C4-O1 at 9.35 °.  Since (I) has no classic hydrogen bonding donors, the crystal packng is stabilized by C—H···O interactions (Table 1)in two directions with aromatic C-H atoms as the donors and  both oxygen atoms of the nitro group and the carbonyl oxygen as the acceptors.  There is also π-stacking along the third direction, the shortest  (*b*), where the aromatic rings are separated by  a unit cell translation of 3.753 (1) Å (Fig. 2).

### Experimental

4-nitrophenyl cyclohexylcarbamate (0.95 g, 3.5 mmol), phenylmethanol (0.40 g, 3.7 mmol) and triethylamine (0.36 g, 3.6 mmol) were reflxued overnight in 20 ml methylene chloride. The solution was washed with 1 N NaOH, water and brine, and then dried with anhydrous Na_2_SO_4_. After removal of the solvent, the product was recovered as a colorless solid (0.5 g). Crystals of (I) were obtained by recrystallization from ethyl acetate as colorless rods.

### Refinement

H atoms were found in difference Fourier maps and subsequently placed in idealized positions with constrained C—H distances of 0.95 Å (C_Ar_H) and 0.98 Å (Csp3H). *U*_iso_(H) values were set to 1.2*U*_eq_ for all H atoms.

### Crystal data

**Table:**

C_10_H_9_NO_4_	*F*_000_ = 864
*M**_r_* = 207.18	*D*_x_ = 1.434 Mg m^−^^3^
Monoclinic, *C*2/*c*	Mo *K*α radiation λ = 0.71073 Å
*a* = 24.491 (6) Å	Cell parameters from 2523 reflections
*b* = 3.753 (1) Å	θ = 1.0–27.5º
*c* = 23.428 (6) Å	µ = 0.11 mm^−^^1^
β = 116.98 (1)º	*T* = 90.0 (2) K
*V* = 1919.0 (9) Å^3^	Thin rod, colorless
*Z* = 8	0.30 × 0.10 × 0.04 mm

### Data collection

**Table:**

Nonius KappaCCD diffractometer	2193 independent reflections
Radiation source: fine-focus sealed tube	1380 reflections with *I* > 2σ(*I*)
Monochromator: graphite	*R*_int_ = 0.049
Detector resolution: 18 pixels mm^-1^	θ_max_ = 27.5º
*T* = 90.0(2) K	θ_min_ = 1.9º
ω scans at fixed χ = 55°	*h* = −31→31
Absorption correction: multi-scan(SCALEPACK; Otwinowski & Minor, 1997)	*k* = −4→4
*T*_min_ = 0.967, *T*_max_ = 0.996	*l* = −30→29
3936 measured reflections	

### Refinement

**Table:**

Refinement on *F*^2^	Secondary atom site location: difference Fourier map
Least-squares matrix: full	Hydrogen site location: inferred from neighbouring sites
*R*[*F*^2^ > 2σ(*F*^2^)] = 0.053	H-atom parameters constrained
*wR*(*F*^2^) = 0.145	*w* = 1/[σ^2^(*F*_o_^2^) + (0.0788*P*)^2^ + 0.0268*P*] where *P* = (*F*_o_^2^ + 2*F*_c_^2^)/3
*S* = 1.04	(Δ/σ)_max_ < 0.001
2193 reflections	Δρ_max_ = 0.32 e Å^−^^3^
137 parameters	Δρ_min_ = −0.28 e Å^−^^3^
Primary atom site location: structure-invariant direct methods	Extinction correction: none

### Special details

**Table:**

Geometry. All e.s.d.'s (except the e.s.d. in the dihedral angle between two l.s. planes) are estimated using the full covariance matrix. The cell e.s.d.'s are taken into account individually in the estimation of e.s.d.'s in distances, angles and torsion angles; correlations between e.s.d.'s in cell parameters are only used when they are defined by crystal symmetry. An approximate (isotropic) treatment of cell e.s.d.'s is used for estimating e.s.d.'s involving l.s. planes.
Refinement. Refinement of *F*^2^ against ALL reflections. The weighted *R*-factor *wR* and goodness of fit *S* are based on *F*^2^, conventional *R*-factors *R* are based on *F*, with *F* set to zero for negative *F*^2^. The threshold expression of *F*^2^ > σ(*F*^2^) is used only for calculating *R*-factors(gt) *etc*. and is not relevant to the choice of reflections for refinement. *R*-factors based on *F*^2^ are statistically about twice as large as those based on *F*, and *R*- factors based on ALL data will be even larger.

### Fractional atomic coordinates and isotropic or equivalent isotropic displacement parameters (Å^2^)

**Table:**

	*x*	*y*	*z*	*U*_iso_*/*U*_eq_	
C1	0.34238 (9)	0.3043 (6)	0.08125 (9)	0.0350 (5)	
H1A	0.3022	0.2636	0.0481	0.042*	
H1B	0.3769	0.2388	0.0753	0.042*	
C2	0.41131 (8)	0.5324 (6)	0.19114 (9)	0.0327 (5)	
H2A	0.4437	0.4551	0.1803	0.049*	
H2B	0.4153	0.4042	0.2293	0.049*	
H2C	0.4150	0.7891	0.1998	0.049*	
C3	0.35044 (8)	0.4545 (6)	0.13665 (9)	0.0258 (5)	
C4	0.29729 (8)	0.5595 (5)	0.14726 (9)	0.0247 (5)	
C5	0.18934 (8)	0.4978 (5)	0.10638 (9)	0.0232 (5)	
C6	0.18535 (8)	0.3892 (5)	0.16081 (8)	0.0248 (5)	
H6	0.2196	0.2858	0.1958	0.030*	
C7	0.13046 (8)	0.4341 (5)	0.16321 (9)	0.0249 (5)	
H7	0.1262	0.3607	0.1998	0.030*	
C8	0.08165 (8)	0.5879 (5)	0.11143 (8)	0.0225 (5)	
C9	0.08549 (8)	0.6948 (5)	0.05692 (8)	0.0239 (5)	
H9	0.0511	0.7963	0.0218	0.029*	
C10	0.14063 (8)	0.6505 (5)	0.05472 (8)	0.0243 (5)	
H10	0.1449	0.7242	0.0182	0.029*	
N1	0.02377 (7)	0.6382 (5)	0.11468 (7)	0.0273 (4)	
O1	0.30028 (5)	0.7338 (4)	0.19146 (6)	0.0309 (4)	
O2	0.24257 (5)	0.4394 (4)	0.09952 (6)	0.0265 (4)	
O3	0.01867 (6)	0.5094 (4)	0.16001 (7)	0.0379 (4)	
O4	−0.01732 (6)	0.8076 (4)	0.07152 (6)	0.0356 (4)	

### Atomic displacement parameters (Å^2^)

**Table:**

	*U*^11^	*U*^22^	*U*^33^	*U*^12^	*U*^13^	*U*^23^
C1	0.0292 (11)	0.0418 (15)	0.0387 (11)	−0.0006 (11)	0.0195 (9)	−0.0047 (11)
C2	0.0274 (11)	0.0333 (14)	0.0390 (12)	0.0015 (10)	0.0165 (10)	−0.0001 (10)
C3	0.0258 (10)	0.0259 (12)	0.0286 (11)	−0.0004 (9)	0.0148 (9)	0.0030 (9)
C4	0.0239 (10)	0.0250 (12)	0.0228 (10)	0.0004 (9)	0.0084 (8)	0.0007 (9)
C5	0.0217 (10)	0.0231 (12)	0.0272 (10)	−0.0010 (9)	0.0133 (8)	−0.0051 (9)
C6	0.0215 (10)	0.0237 (12)	0.0246 (10)	−0.0001 (9)	0.0066 (8)	−0.0014 (9)
C7	0.0258 (10)	0.0254 (12)	0.0238 (10)	−0.0008 (9)	0.0116 (8)	−0.0005 (8)
C8	0.0199 (9)	0.0233 (12)	0.0251 (10)	−0.0017 (9)	0.0107 (8)	−0.0032 (9)
C9	0.0230 (10)	0.0233 (12)	0.0219 (9)	0.0006 (9)	0.0070 (8)	−0.0013 (9)
C10	0.0269 (10)	0.0248 (12)	0.0204 (9)	0.0004 (9)	0.0102 (8)	−0.0007 (9)
N1	0.0238 (9)	0.0312 (11)	0.0268 (9)	0.0008 (8)	0.0113 (7)	−0.0005 (8)
O1	0.0260 (7)	0.0375 (10)	0.0295 (7)	−0.0029 (7)	0.0129 (6)	−0.0088 (7)
O2	0.0201 (7)	0.0343 (8)	0.0258 (7)	−0.0005 (6)	0.0111 (6)	−0.0043 (6)
O3	0.0317 (8)	0.0518 (11)	0.0365 (8)	0.0040 (7)	0.0210 (7)	0.0094 (7)
O4	0.0251 (7)	0.0489 (11)	0.0319 (7)	0.0098 (7)	0.0121 (6)	0.0059 (7)

### Geometric parameters (Å, °)

**Table:**

C1—C3	1.345 (3)	C5—O2	1.402 (2)
C1—H1A	0.9500	C6—C7	1.381 (3)
C1—H1B	0.9500	C6—H6	0.9500
C2—C3	1.487 (2)	C7—C8	1.385 (3)
C2—H2A	0.9800	C7—H7	0.9500
C2—H2B	0.9800	C8—C9	1.382 (2)
C2—H2C	0.9800	C8—N1	1.466 (2)
C3—C4	1.485 (3)	C9—C10	1.385 (3)
C4—O1	1.199 (2)	C9—H9	0.9500
C4—O2	1.375 (2)	C10—H10	0.9500
C5—C10	1.381 (3)	N1—O3	1.224 (2)
C5—C6	1.384 (3)	N1—O4	1.231 (2)
			
C3—C1—H1A	120.0	C7—C6—H6	120.7
C3—C1—H1B	120.0	C5—C6—H6	120.7
H1A—C1—H1B	120.0	C6—C7—C8	119.00 (17)
C3—C2—H2A	109.5	C6—C7—H7	120.5
C3—C2—H2B	109.5	C8—C7—H7	120.5
H2A—C2—H2B	109.5	C9—C8—C7	122.47 (17)
C3—C2—H2C	109.5	C9—C8—N1	118.91 (16)
H2A—C2—H2C	109.5	C7—C8—N1	118.62 (16)
H2B—C2—H2C	109.5	C8—C9—C10	118.42 (17)
C1—C3—C4	121.11 (17)	C8—C9—H9	120.8
C1—C3—C2	124.19 (18)	C10—C9—H9	120.8
C4—C3—C2	114.70 (17)	C5—C10—C9	119.11 (17)
O1—C4—O2	122.47 (17)	C5—C10—H10	120.4
O1—C4—C3	125.08 (17)	C9—C10—H10	120.4
O2—C4—C3	112.45 (16)	O3—N1—O4	123.34 (16)
C10—C5—C6	122.41 (17)	O3—N1—C8	118.44 (15)
C10—C5—O2	116.30 (16)	O4—N1—C8	118.22 (15)
C6—C5—O2	121.19 (16)	C4—O2—C5	118.00 (14)
C7—C6—C5	118.58 (17)		
			
C1—C3—C4—O1	−170.1 (2)	C6—C5—C10—C9	−0.5 (3)
C2—C3—C4—O1	9.4 (3)	O2—C5—C10—C9	176.01 (16)
C1—C3—C4—O2	8.8 (3)	C8—C9—C10—C5	0.8 (3)
C2—C3—C4—O2	−171.74 (17)	C9—C8—N1—O3	172.43 (18)
C10—C5—C6—C7	0.3 (3)	C7—C8—N1—O3	−7.5 (3)
O2—C5—C6—C7	−176.06 (18)	C9—C8—N1—O4	−7.5 (3)
C5—C6—C7—C8	−0.4 (3)	C7—C8—N1—O4	172.65 (18)
C6—C7—C8—C9	0.8 (3)	O1—C4—O2—C5	−5.8 (3)
C6—C7—C8—N1	−179.34 (17)	C3—C4—O2—C5	175.30 (15)
C7—C8—C9—C10	−1.0 (3)	C10—C5—O2—C4	129.61 (19)
N1—C8—C9—C10	179.14 (16)	C6—C5—O2—C4	−53.8 (2)

### Hydrogen-bond geometry (Å, °)

**Table:**

*D*—H···*A*	*D*—H	H···*A*	*D*···*A*	*D*—H···*A*
C7—H7···O1^i^	0.95	2.41	3.130 (2)	133
C1—H1B···O4^ii^	0.95	2.64	3.546 (3)	159
C2—H2A···O3^ii^	0.98	2.68	3.611 (2)	159
C9—H9···O4^iii^	0.95	2.46	3.282 (2)	145

Symmetry codes: (i) −*x*+1/2, *y*−1/2, −*z*+1/2; (ii) *x*+1/2, *y*−1/2, *z*; (iii) −*x*, −*y*+2, −*z*.
